# Meta-learning prediction of physical and chemical properties of magnetized water and fertilizer based on LSTM

**DOI:** 10.1186/s13007-021-00818-2

**Published:** 2021-11-24

**Authors:** Jing Nie, Nianyi Wang, Jingbin Li, Kang Wang, Hongkun Wang

**Affiliations:** 1 College of Mechanical and Electrical Engineering, Shihezi University, Xinjiang, China; 2 Key Laboratory of Modern Agricultural Machinery of Xinjiang Production and Construction Corps, Shihezi, China

**Keywords:** Meta-learning, Regression prediction, Meta-learner LSTM, MAML

## Abstract

**Background:**

Due to the high cost of data collection for magnetization detection of media, the sample size is limited, it is not suitable to use deep learning method to predict its change trend. The prediction of physical and chemical properties of magnetized water and fertilizer (PCPMWF) by meta-learning can help to explore the effects of magnetized water and fertilizer irrigation on crops.

**Method:**

In this article, we propose a meta-learning optimization model based on the meta-learner LSTM in the field of regression prediction of PCPMWF. In meta-learning, LSTM is used to replace MAML’s gradient descent optimizer for regression tasks, enables the meta-learner to learn the update rules of the LSTM, and apply it to update the parameters of the model. The proposed method is compared with the experimental results of MAML and LSTM to verify the feasibility and correctness.

**Results:**

The average absolute percentage error of the meta-learning optimization model of meta-learner LSTM is reduced by 0.37% compared with the MAML model, and by 4.16% compared with the LSTM model. The loss value of the meta-learning optimization model in the iterative process drops the fastest and steadily compared to the MAML model and the LSTM model. In cross-domain experiments, the average accuracy of the meta-learning optimized model can still reach 0.833.

**Conclusions:**

In the case of few sample, the proposed model is superior to the traditional LSTM model and the basic MAML model. And in the training of cross-domain datasets, this model performs best.

## Background

In modern agriculture, magnetic treatment of water and fertilizer irrigation has been the underlying physical technology to increase crop yields [1]. Some studies have confirmed that irrigation with magnetized water and fertilizer can enhance the ability of some crops to absorb nutrients [1, 2], and also improve some specific goals [3–5]. In fact, the essence of magnetized water and fertilizer is that under the action of a magnetic field, its physical and chemical properties, such as surface tension coefficient, viscosity, conductivity, PH value, will change [6–8], this has an impact on the growth of crops. This article hopes to predict the physical and chemical properties of magnetized water and fertilizer (PCPMWF), further help to explore its impact on crop irrigation.

At present, with the development of smart agriculture, the application of deep learning in agriculture has been extensively studied [9, 10]. For example, two deep learning architectures are used to identify and predict pests and diseases based on features such as texture and color [11, 12], and methods such as DBN and CNN are used to distinguish different crops and weeds by identifying leaves, so as to optimize the implementation of herbicides [13–16]. Not only that, with the development of deep learning structures such as recurrent neural networks (RNN) and long short-term memory networks (LSTM), the forecasting and early warning of time series data has also developed rapidly [17, 18]. It has application potential in the field of intelligent management of agricultural production.

Although deep learning has achieved advanced results and often has high accuracy, at the same time, the success of deep neural network models depend chiefly on a large number of samples and multiple iterations of training parameters [19–21], leading to this is not applicable in some research fields. Especially, collecting large-scale data will bring a serious burden, because the collection of data often requires a lot of workforce and time cost [22], and when the number of samples with supervised information is too small, the training of deep learning models is prone to overfitting [19, 23]. The overfitting problem may cause the loss function to be very close to 0, and the model fits all the data in the training set as much as possible, but there is not enough data to restrict it to better generalize to new samples.

For this reason, one way is to use more complex networks and larger datasets [24, 25]. However, the community, which is in the bottleneck of deep learning, has to stand on another new starting point to break through the difficulties. The solution of how to train a model with a small amount of data has attracted the attention of some scholars, and they have proposed a few-shot learning method. At this stage, few-shot learning methods are divided into three categories: data enhancement, metric learning, and meta-learning [23]. The successful application of few-shot learning in other fields has caused it to gain more and more attention in the agricultural field, including plant segmentation, pests and diseases identification, remote sensing, crop status assessment, etc. Notably, Li et al. used CNN feature extractor to train a few samples through triplet loss to distinguish different pest species [26]. Furthermore, Li et al. proposed a semi-supervised few-shot learning method for plant leaf disease identification, which verified its correctness and generalization [24]. Azam Karami et al. explored the application of few-shot learning in remote sensing technology for automatic plant counting and positioning [27]. Wang et al. proposed a few-shot learning method based on the Siamese network to solve a leaf classification problem with a small sample size [28]. The above related studies are all in the case of unable to obtain enough samples, learning through a few samples to solve practical problems, reducing the number of samples and cost. In essence, few-shot learning mines the high-level semantic representation of things, that is, extract and disseminate prior knowledge from the task set, so that the trained model can be transferred, and the influence of the learned experience on the completion of new tasks is applied to a few samples.

Starting from the research direction of this article, we are more inclined to use few-shot learning methods. Mainly for the “large-scale datasets” problem, there are two difficulties in this research: First of all, because the research on PCPMWF in the agricultural field is not comprehensive, there is no data related to magnetized water and the physical and chemical parameters of water and fertilizers in the public datasets, which requires us to collect data through experiments. What's more, the magnetization experiment and the measurement steps of the parameters are very cumbersome, the calculation process is complicated, and the parameters of the water and fertilizer solution are in a flowing state during irrigation are difficult to determine, which makes data collection time-consuming and laborious. Altogether, collecting large-scale datasets is difficult and costly. The few-shot learning method is hopeful to bring important value and significance to this research, and it has broad development potential in the agricultural field.

In this paper, we propose a meta-learning optimization model using LSTM as the meta-learner to apply to the field of parameter prediction field of PCPMWF. This method replaces the traditional gradient descent learner of MAML with a long and short-term memory network in meta-learning to process regression tasks. We conducted water and fertilizer magnetization experiments to collect samples, summarized and compared the pros and cons of LSTM, MAML, and optimization models. Not only did the meta-learning MAML and deep-learning LSTM comparative experiments on the datasets ensure the feasibility of few-shot learning, but also further improved the meta-learning method, and also verified the superiority of the optimization model based on the LSTM meta-learner through experimental comparisons. Finally, a better method is proposed for the prediction of the physical and chemical properties of magnetized water and fertilizers, which can provide some references for further research in this direction in the future.

The contributions of this work are three-fold:

1. We collected samples of PCPMWF through experiments for research in the field of agricultural irrigation magnetized water and fertilizer.
2. We propose a meta-learning optimization model using LSTM as the meta-learner to predict PCPMWF.
3. We compare the proposed method with the experimental results of MAML and LSTM to verify the feasibility and correctness of the method in the field of parameter prediction of PCPMWF.

## Materials

The goal task is to predict the changing trend of PCPMWF under the condition of irrigation as the intensity of the magnetic field increases. The magnetic field strength increases regularly from 0 to 450mT with an arithmetic difference of 50mT, then each physical and chemical property parameter is a series of sequence data indexed by the magnetic field strength.

As mentioned above, since the water and fertilizer solution is in a flowing state during irrigation, its physical and chemical properties are difficult to determine, and the parameters of the magnetized static water and fertilizer solution are relatively easy to collect. The datasets we collect are divided into “dynamic” and “static” categories. The specific data are collected by the water and fertilizer in the flowing or static state of the magnetization device, including surface tension coefficient $\sigma$ ($N/m$), viscosity $\eta$ ($mPa \cdot S$), conductivity EC($\mu S/cm$)and PH value four physical and chemical properties parameters. To this end, we set up a magnetizer test bench. After the water and fertilizer solution is magnetized, the final required data is obtained through precision instrument measurement and complex calculations. The specific measurement methods of each parameter are as follows:

- (1) Use the pull-off method to measure the surface tension coefficient. Using the liquid surface tension coefficient measuring instrument, the reading value of the digital voltmeter immediately before the ring liquid film is broken and the reading value of the digital voltmeter after the breaking are $U_{1}$ and $U_{2}$, respectively. Suppose the inner and outer diameters of the hoisting ring are $D_{1}$ and $D_{2}$, and the conversion coefficient $K$ of the instrument measured by the stepwise difference method is brought into the formula, expressed as Eq. (1):
  1 $$\sigma = \frac{{U_{1} - U_{2} }}{{K\pi \left( {D_{1} + D_{2} } \right)}}$$
- (2) Use the falling ball method to measure the viscosity. The measuring tool is a small ball and a cylinder containing a magnetized water and fertilizer liquid. Set the density of the ball as $\rho$, the density of the magnetized water and fertilizer as $\rho_{0}$, the diameter of the ball as $d$, the inner diameter of the cylinder as $D$, and the depth of the liquid as $H$. In addition, the photoelectric gate is used to measure and calculate that the uniform drop speed of the ball in the liquid is $v$, which is brought into the formula, expressed as Eq. (2):
  2 $$\eta = \frac{{(\rho - \rho_{0} )gd^{2} }}{{18v[(1 + 2.4\frac{d}{D})(1 + 3.3\frac{d}{2H})]}}$$
- (3) Measure conductivity with the water quality test pen.
- (4) Measure the PH value with the water quality test pen.

The total number of dynamic magnetized water and fertilizer data collected in this study is 64, and the total number of static magnetized water and fertilizer data is 424 (hereinafter referred to as “dynamic data” and “static data”). The data details are shown in Table 1.

**Table 1:** The details of dynamic data and static data

Physical and chemical properties Parameters	$\sigma$	$\eta$	EC	PH value
Dynamic Data/Piece	16	16	16	16
Static Data/Piece	106	106	106	106

The datasets are divided into the training set and the test set, and they are divided into support set and query set. The two datasets are disjoint. In the research of this article considering cross-domain factors, the support set is the static magnetized water and fertilizer dataset. The query set is a very small amount of dynamic magnetized water and fertilizer dataset. Different from the classification task (N-way K-shot classification problem), in the regression task, each task is no longer a batch of the classification task, but all the data is sent to the network, and a forward calculation and reverse calculation are completed.

## Methods

### MAML algorithm

Few-shot regression is to learn generalization through very few or fewer training samples, and the robust regressor can maintain high accuracy and scalability to predict new data. As the most extensive meta-learning algorithm, MAML's basic idea is to find an optimal initial parameter, which can quickly learn new regression tasks with fewer gradient steps than deep learning. In MAML training, starting from initializing the optimal weights, the gradient descent method is repeatedly used to find the optimal weights, that is, to minimize the loss to train the network, so as to achieve convergence.

**Fig. 1:**
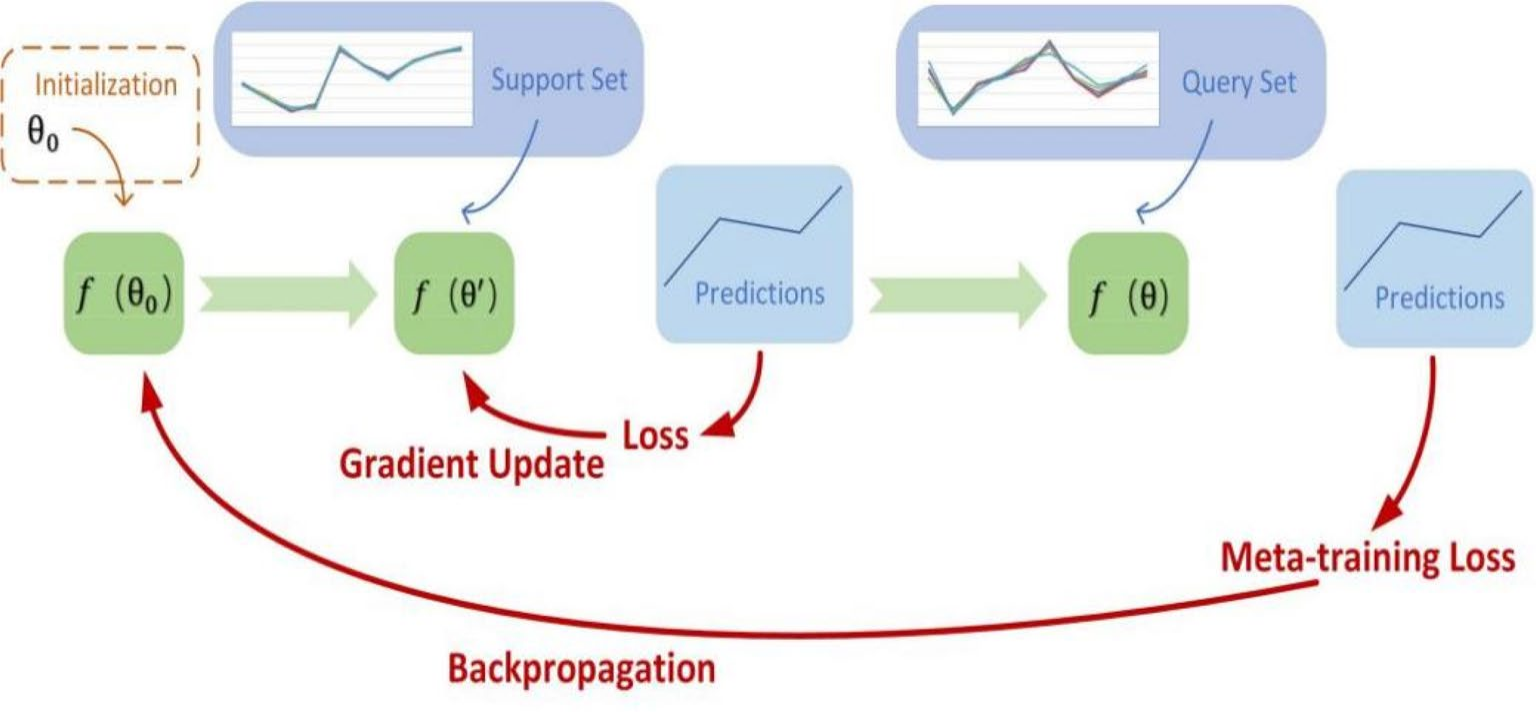
MAML solution

In this work, the overall solution of MAML is shown in Fig. 1. The structure covers inner and outer loops. The inner loop is used for update, that is, calculate the loss and update the gradient in each task, and find the optimal parameter $\theta_{i}^{{\prime}}$ of each task. The outer loop is used for backpropagation, that is, in each new task, the randomly initialized model parameter $\theta_{i}^{{\prime}}$ is updated by calculating the gradient relative to $\theta$ obtained in the inner loop.

Specifically, firstly, the weight $\theta = \theta_{0}$ needs to be initialized, then gradient descent is used in each task to update $\theta_{i}^{{\prime}}$ for this specific task, and then the original parameter $\theta_{i}^{{\prime}}$ is updated relative to $\theta$, which realizes the use of gradient descent to minimize the loss training regression. The network gets the optimal weight. This reflects the obvious difference between meta-learning and deep learning. Deep learning is to update the same parameter $\theta$ according to different batches of tasks, and the result of training will get a global (or for each task) optimal solution, but this may not be the optimal solution for a certain task. Meta-learning is different, it does not update the parameter $\theta$ for each task, but updates the parameter $\theta$ through the $\theta_{i}^{{\prime}}$ of each task. In this way, the possible performance of $\theta$ on each task is not the best, but it can be guaranteed that the parameter $\theta$ is the most "sensitive". In other words, it is very sensitive to new tasks, and small changes in parameters can make a big change in loss. Therefore, a small amount of data can be used to complete the training of new tasks [29].

According to the foregoing, the extracted data points are divided into support set and query set, the support set is used to find the optimal parameter $\theta_{i}^{{\prime}}$ in the inner loop, and the query set is used to find the optimal parameter $\theta$ in the outer loop.

For regression tasks, the method uses mean square error (MSE) as the loss function:

3 $$L_{{T_{i} }} (f_{\theta } ) = \sum\limits_{{x_{j} ,y_{j} \sim T_{i} }} {\left\| {f_{\theta } (x_{i} ) - y_{i} } \right\|_{2}^{2} }$$

The pseudo-code of the few-sample regression problem is shown in Table 2.

**Table 2:** Algorithm for updating model parameters

**Algorithm 1** Meta-Learning Regression Model
**Input:** MAML with parameters $$\theta$$, Base-Learner with step size hyperparameter $$\alpha$$, Meta-Learner with step size hyperparameter $$\beta$$ $$\theta_{0}$$$$\leftarrow$$ random initialization #Randomly initialize model parameters
**while** not done **do** $$D_{train}$$, $$D_{test}$$$$\leftarrow$$ random datasets **for all** $${\text{T}}_{{\text{i}}}$$ **do** $$x_{j}$$, $$y_{j}$$$$\leftarrow$$ random batch from support set $$L \leftarrow L_{{T_{i} }} \left( {f_{\theta } } \right)$$#Get loss using Eq. (3) $$g \leftarrow \nabla_{\theta } L_{{T_{i} }} \left( {f_{\theta } } \right)$$#Evaluate gradient with respect to parameters $$\theta$$ $$\theta_{i}^{{\prime}} \leftarrow \theta - \alpha \nabla_{\theta } L_{{T_{i} }} \left( {f_{\theta } } \right)$$#Update Meta-Learner parameters **end for** $$X$$, $$Y$$← random batch from quest set Update $$\theta \leftarrow \theta - \theta_{i}^{{\prime}} \leftarrow \beta \nabla_{\theta } \sum\limits_{{T_{i} \sim P(T)}} {L_{{T_{i} }} \left( {f_{{\theta_{i}^{{\prime}} }} } \right)}$$ #Update Base-Learner parameters **end while**

### Meta-learner LSTM

As known, LSTM, as a special RNN, solves the problem of gradient disappearance and gradient explosion of ordinary RNN in the long sequence data training process. In this work, we propose to use LSTM as a meta-learner to optimize meta-learning to perform regression tasks, replace the traditional gradient descent method with a long and short-term memory network as a learner, and store these updated sequences in LSTM, called meta-learner LSTM. Therefore, the role of LSTM in the optimization model is particularly important.

**Fig. 2:**
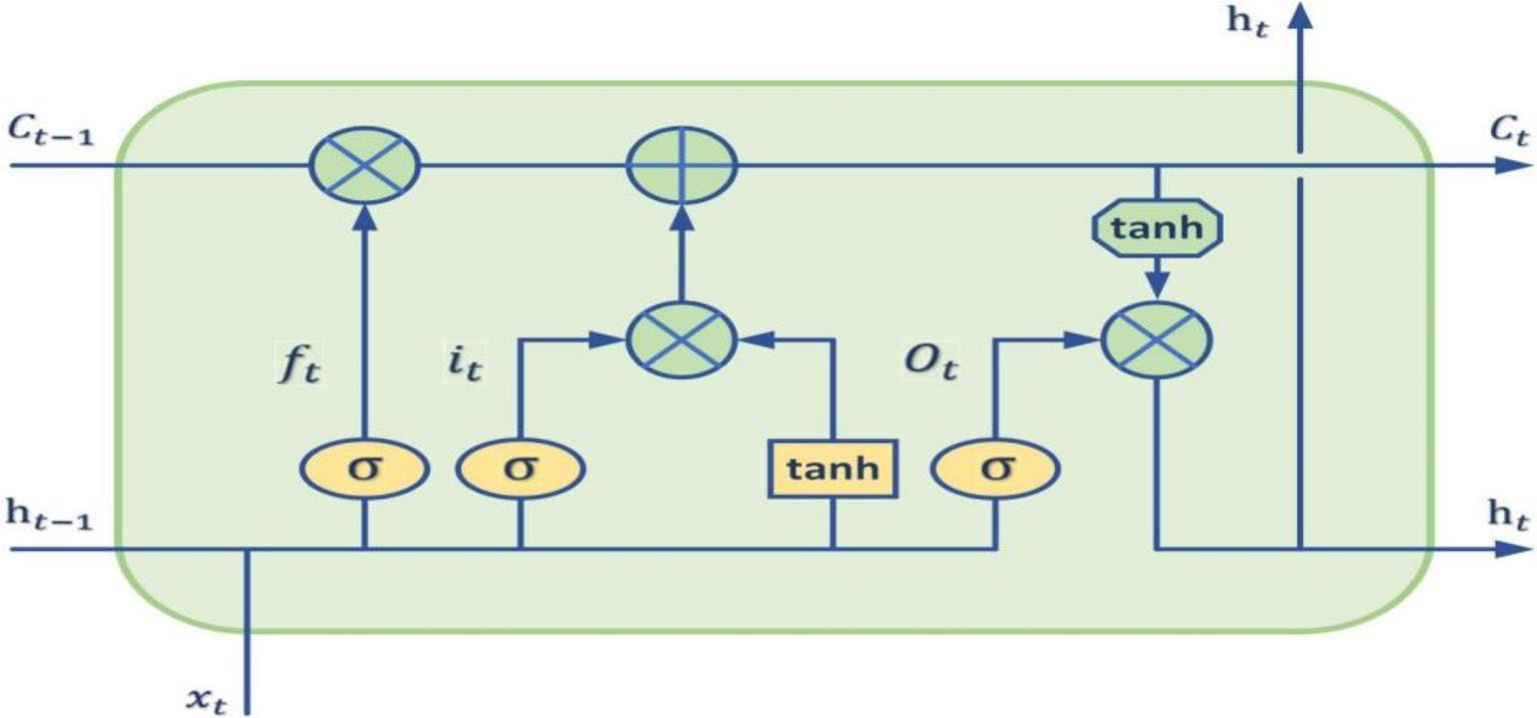
Internal structure of LSTM block

There are only simple neurons in the cyclic neural network, and LSTM blocks are used to replace neurons in LSTM to train long-term dependent information. The LSTM block is shown in Fig. 2.

First, the cell state $C_{t - 1}$ of the previous layer is multiplied by the forgetting vector $f_{t}$ point by point (denoted as $\odot$). If it is multiplied by a value close to 0, then in the new cell state, this information needs to be discarded. Then add this value to the output value $i_{t} \odot \tilde{C}_{t}$ of the input gate point by point, and update the new information found by the neural network to the current cell state $C_{t}$. Finally, the updated cell state is obtained. So far, the cell state update equation is derived, expressed as Eq. (4):

4 $$C_{t} = f_{t} \odot C_{t - 1} + i_{t} \odot \tilde{C}_{t}$$

**Fig. 3:**
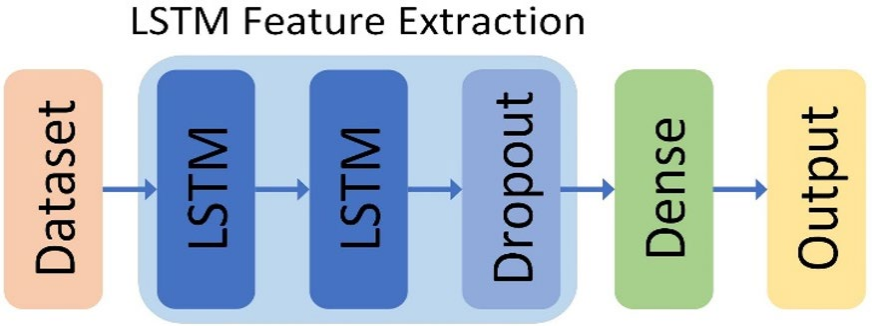
Meta-learner LSTM model structure

However, in meta-learner LSTM, the LSTM block is a neural network structure as a recurrent layer. Using the LSTM block alone cannot build a complete learner. It is necessary to build the LSTM network and the dense layer. Meta-learner LSTM uses a two-layer LSTM network. The main reason for stacking LSTM layers is to allow greater learner complexity. In the case of a simple feedforward network, stack LSTM layers to create a hierarchical feature representation of the input data. Used for learning tasks. Of course, double-edged knives can also lead to over-adaptation and poor performance. As far as this structure is concerned, it is sufficient to improve from a single-layer LSTM layer to a two-layer LSTM layer. At the same time, cooperate with the Dropout layer to avoid the over-fitting phenomenon due to the excessive number of hidden layer nodes [30]. Place the Dense layer and finally process the previously extracted features with nonlinear changes, and sort out the weights in the LSTM network [31]. The model structure is shown in Fig. 3

### Meta-learning optimization model

It can be seen from the above that the essence of MAML is to use gradient descent to learn the optimal initial parameter values. This paper proposes an optimization model that hopes that the meta-learner optimizes itself through gradient descent. After clever replacement, it becomes the use of LSTM's state update formula to update the model parameters. Therefore, the purpose of Meta-Learner is to learn the update rules of LSTM and apply them to the parameters of the update model, so as to better “learning to learn”.

#### Overall framework

**Fig. 4:**
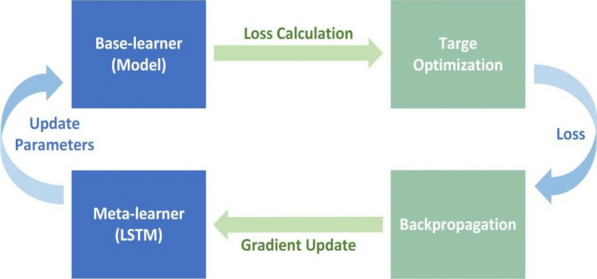
The overall framework of the meta-learning optimization model

If we call meta-learner LSTM an optimizer, then Base-learner is called an optimization object. The overall framework of the meta-learning optimization model is shown in Fig. 4, which shows that the cyclical framework of the optimization model consists of four parts.

In step 1, use LSTM to find the optimal parameters and send them to the model to update the model parameters.

In step 2, the model then uses the new parameters of step 1 to calculate the loss.

In step 3, when the step 2 achieves the goal optimization, the loss is backpropagated to the LSTM.

In step 4, calculate the gradient based on the loss of step 3 to optimize the LSTM itself, and further update the model parameters.

We can see that although the optimization model uses LSTM instead of the gradient descent method to find the optimal parameters, the gradient descent method needs to be used to optimize the LSTM, so the gradient descent method is still an indispensable part. In other words, the optimization model learning uses meta-learner LSTM to perform gradient descent, and meta-learner LSTM is optimized by gradient descent. From this point of view, the gradient descent method can be regarded as an update sequence from the output layer to the input layer. The LSTM cell update equation (expressed as Eq. (4)) corresponds to the update equation of the gradient descent, expressed as Eq. (5).

5 $$\theta_{t} = \theta_{t - 1} - \alpha_{t} \nabla_{{\theta_{t - 1} }} L_{t}$$

When $$f_{t} = 1$$,

6 $$C_{t - 1} = \theta_{t - 1}$$

7 $$i_{t} = \alpha_{t}$$

8 $$\tilde{C}_{t} = \nabla_{{\theta_{t - 1} }} L_{t}$$

Analyze the specific role of the LSTM block in the meta-learning optimization scenario with reference to Fig. 2. The role of forget gate ($f_{t}$) in the optimization model is particularly important. When the loss is large and the gradient is close to zero, the cell state is selectively forgotten, and it is decided to discard the parameter value $\theta_{t - 1}$ and loss $L_{t}$ that cause a large loss from the cell state, and its gradient $L_{t}$, thereby shrinking the model parameters. Input gate ($i_{t}$) determines the rest of the new information to enter the cell state, so it can determine the value of the updated model. It is used to adjust the learning rate $\alpha_{t}$, which can prevent the network model from diverging and quickly learn. The final output gate ($O_{t}$) selectively outputs updated information $\theta_{t}$ based on the current cell state.

#### Comparison of model structures

**Fig. 5:**
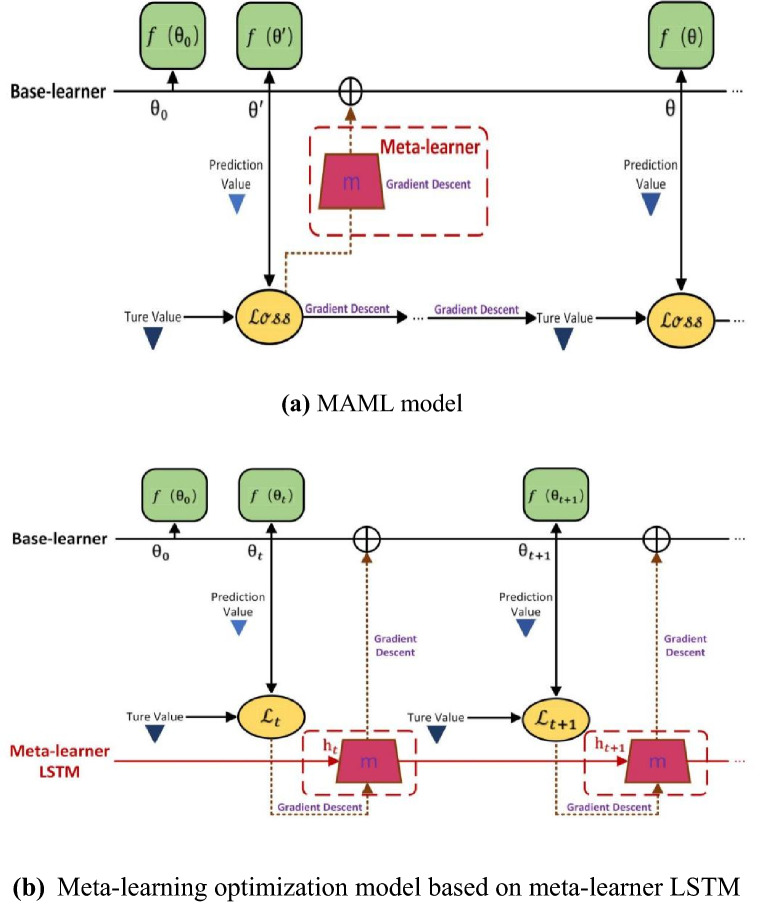
Comparison of the structure of the two models. **a** MAML model, **b** Meta-learning optimization model based on meta-learner LSTM

Knowing the internal operating mechanism of meta-learner LSTM, we further compare the meta-learning optimization model of meta-learner LSTM with the MAML model, as shown in Fig. 5, to understand how meta-learner LSTM performs optimization in the model structure.

With respect to the static model MAML gradient descent as meta-learner, meta-learning optimization model meta-learner LSTM $h_{t}$ hidden updated over time. It has its own parameters, so suitable parameters can be found to minimize the loss through gradient descent. It is worth noting that when training on the support set, its parameters will not change. Instead, its parameters will be updated with gradient descent relative to the loss on the query set. It takes the gradient of the loss function relative to $\theta_{t}$ as the input, this serves as an update to the meta-learner LSTM (optimizer) itself, it is trained and calculated to minimize the loss. Furthermore, the gradient descent of the loss function is sent to the optimization object and added to $\theta_{t}$ to become $\theta_{t + 1}$, that is updated when the model is in the next state (time t + 1).

## Results

In this section, the meta-learning optimization model based on meta-learner LSTM is used to conduct comparative experiments with LSTM and MAML model predictions, and the experimental result data of Mean Absolute Error (MAE) and Mean Absolute Percentage Error (MAPE) are obtained, fitting of the true value, predicted value and iteration of the loss function. And in the cross-domain situation, the advantages and disadvantages of the meta-learner LSTM meta-learning optimization model and the MAML model are compared. The experimental hardware and software environments are the NVIDIA GeForce RTX 2080 with 32 GB memory and the libraries of Python (version 3.7.11), Pytorch (version 1.9.0), Numpy (version 1.21.2), and Pandas (version 1.3.2).

In order to ensure a large comparison, the model parameters and dataset will be adjusted to be consistent. The specific parameters are shown in Table 3.

**Tab 3:** Model-specific parameters and explanation

Model parameters	Explanation	LSTM Model	MAML Model	Optimization Model
num_epochs	Number of iterations	20	20	20
update_lr	Learning rate	0.005	0.005	0.005
dropout	Random inactivation rate	0.5	null	0.5
hidden_size	LSTM hidden layer size	256	null	256
num_layers	Number of LSTM layers	2	null	2

It should be noted that the number of iterations is unified to 20. The learning rate of the optimization model is divided into two types: inner loop and outer loop, both of which are 0.005. For the MAML model, there is no LSTM part, so the remaining three parameter values are null, and for the optimization model, these three parameter values refer to its meta-learner LSTM parameters.

### Results of model prediction

We have collected a total of 64 dynamic magnetized water and fertilizer data, and a total of 424 static magnetized water and fertilizer data. Considering that LSTM cannot be trained with cross-domain data, this group of experiments uniformly used single-domain “static data” to train three models, and selected 100 of the “surface tension coefficient” as the total dataset. The distribution of the dataset is shown in Table 4.

**Table 4:** Specific distribution of dataset

Models	Total dataset	Training set		Test set	
LSTM Model	100	90		10	
MAML Model and Optimization Model	100	Support set	Quest set	Support set	Quest set
		40	10	10	5

Since the LSTM model is different from the meta-learning model, the dataset only needs to be divided into the training set and the test set according to 0.9:0.1, and the MAML model and optimization model is used as the meta-learning model. Specifically, after the dataset is divided into training set and test set according to 0.8:0.2, further random samples will be drawn from it to be used as support set and query set. Therefore, we set to select 40 support sets and 10 query sets in the 80 training sets, and select 10 support sets and 5 query sets in the 20 test sets.

Under the above experimental conditions, use MAE and MAPE to describe the prediction results of the model expressed as Eq. (9) and Eq. (10). Among them, $y_{i}$ represents the true value, and $\hat{y}_{1}$ represents the predicted value of the model. When the predicted value is completely consistent with the true value, MAE is equal to 0, that is, a perfect model; the greater the error, the greater the value. Similarly, a MAPE of 0% indicates a perfect model, and a MAPE greater than 100% indicates an inferior model.

9 $$MAE = \frac{1}{n}\sum\limits_{i = 1}^{n} {\left| {\hat{y}_{1} - y_{i} } \right|}$$

10 $$MAPE = \frac{100\% }{n}\sum\limits_{i = 1}^{n} {\left| {\frac{{\hat{y}_{1} - y_{i} }}{{y_{i} }}} \right|}$$

Based on the model parameters that have been set above and the dataset training model, the specific experimental data obtained are shown in Table 5. We can see that in the case of very few samples, the final MAE and MAPE obtained by the LSTM model are 0.22% and 4.69%, respectively, which are significantly higher than the experimental results of the meta-learning model. Although the prediction effect is similar, it can be concluded that the MAE and MAPE of the optimization model are slightly lower than that of the MAML model. Its prediction effect is the best among the three models.

**Table 5:** Comparison of MAE and MAPE of prediction results

Models	MAE	MAPE
LSTM Model	0.22%	4.69%
MAML Model	0.05%	0.90%
Optimization Model	0.02%	0.53%

**Fig. 6:**
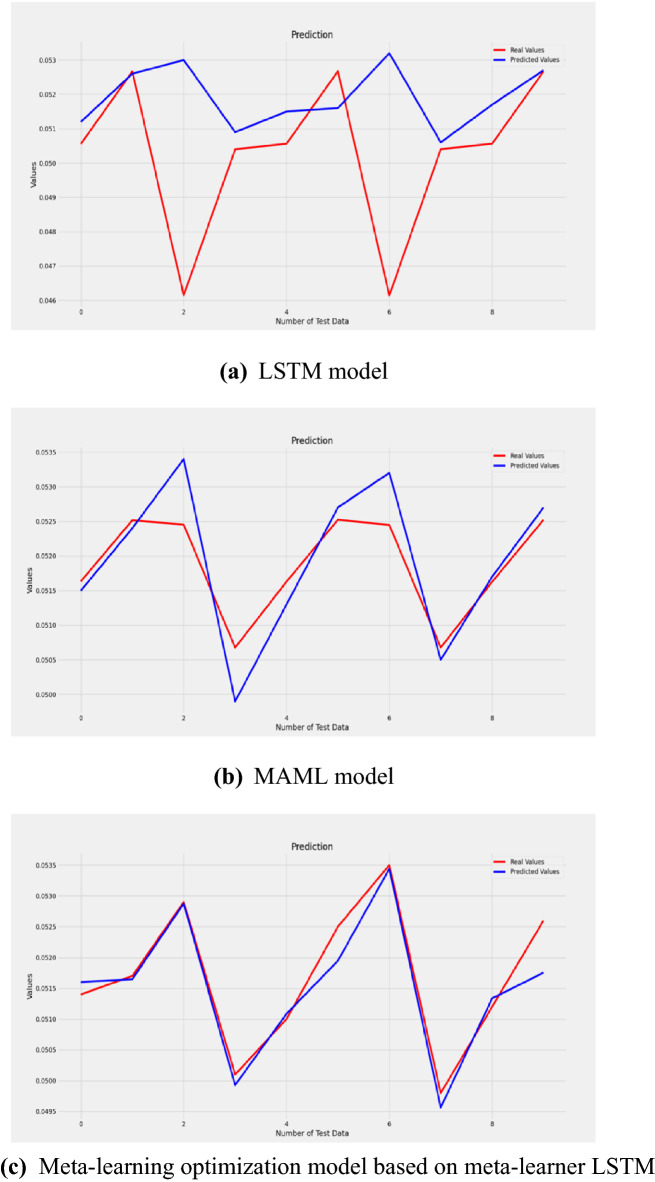
Fitting curve of true value and predicted value. (LSTM model, **b** MAML model, **c** Meta-learning optimization model based on meta-learner LSTM

The model predicts the last 4 sets of data from the first 6 sets of data, that is, through training the physical and chemical property parameters when the magnetization is 0-250mT to predict the properties of the physical and chemical parameters when the magnetization is 300-450mT. The fitting effect of the true value and the predicted value is intuitively reflected in Fig. 6. The test set randomly selects 10 sample data of the surface tension coefficient of the seventh group (that is, in the case of 300 mT magnetization) in the dataset. The red line represents the actual value of the surface tension coefficient, and the blue line represents the predicted value of the surface tension coefficient. It can be seen from the real value that the surface tension coefficient is quite random and is an unstable sequence. Hence the fitting effect of the LSTM model is not very good, while the fitting curve of the optimization model is relatively more suitable.

### Results of the iterative loss function

**Fig. 7:**
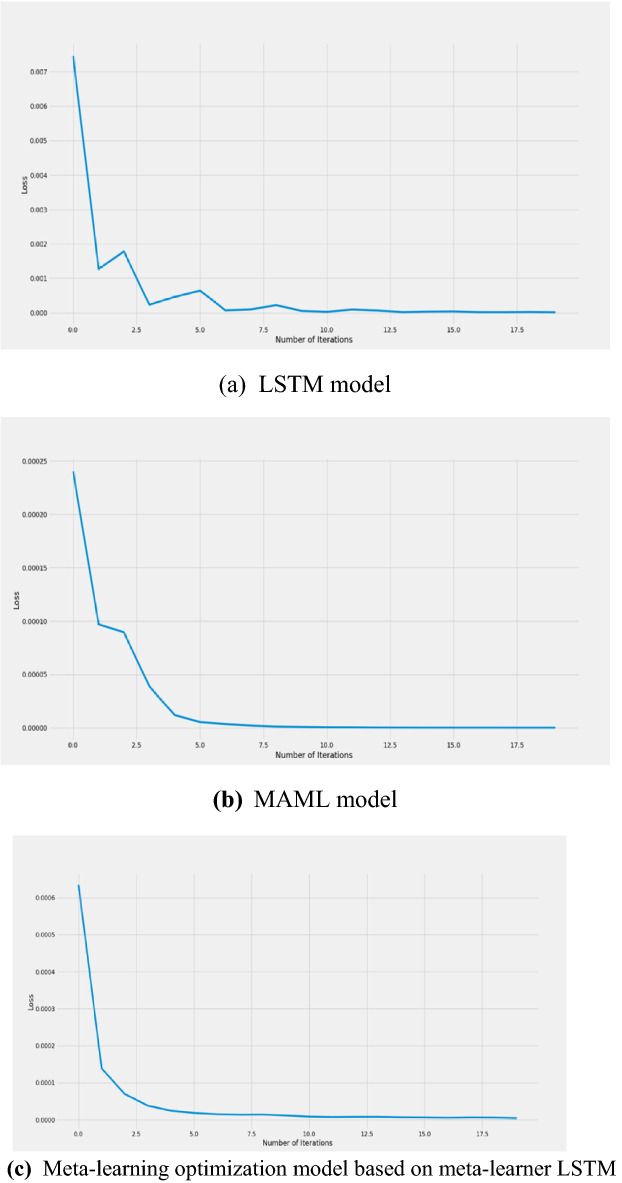
Loss function curve. **a** LSTM model, **b** MAML model, **c** Meta-learning optimization model based on meta-learner LSTM

Loss function is used to estimate the degree of inconsistency between the predicted value of the network model and the true value. With continuous iteration, the smaller the loss function, the better the robustness of the model. This experiment uses the common mean square error loss in regression problems, and the loss function curves of the three models are shown in Fig. 7.

It can be seen from the figure that the losses of the three models in the initial iterations are quite different. In particular, the loss value of the LSTM model can reach 0.007 and above. Compared with the loss value of the two models of the meta-learning algorithm. The accuracy is up to one decimal place. Knowing that the epoch is set to 20, their loss values have stabilized at around 10 iterations. Only the LSTM model fluctuates slightly after 10 iterations, and shows a trend of “oscillation attenuation” before stabilizing. In contrast, the loss function of the optimization model is the smoothest, and the decline is the fastest, reflecting its robustness. The decrease of the loss value of the MAML model slowed down between 1 and 2 iterations, which highlights the optimization effect of the optimization model.

### Results of cross-domain datasets experiment

**Fig. 8:**
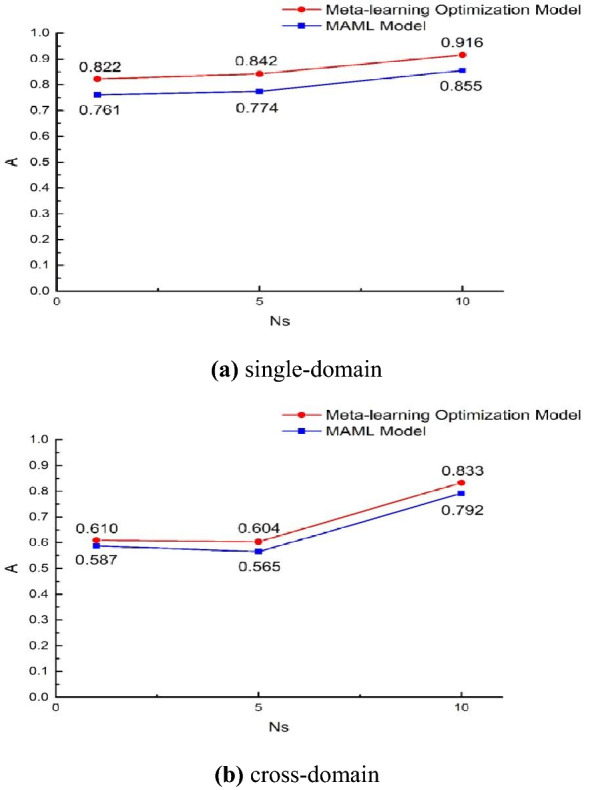
The average accuracy of the model in single-domain and cross-domain cases. **A **single-domain, **b** cross-domain

The previous article only uses “static data” to verify the MAE, MAPE and loss functions of the three models, which belong to a “single-domain” experiment. Now consider a more complex data set and compare it with the “cross-domain” experiment. The cross-domain in this experiment refers to the training and prediction of “dynamic data” from “static data”, that is, static data as a support set and dynamic data as a query set. The average accuracy results of the model in the single domain and cross domain are shown in Fig. 8. In the figure, the ordinate is the average accuracy $A$, and the abscissa is the support set capacity $N_{s}$ during model training, taking 1, 5, and 10 respectively, and the query set capacity $N_{q}$ during training is a fixed value of 5.

Comparing the two figures, the average accuracy of the optimized model is always higher than that of the MAML model regardless of single-domain or cross-domain experiments. By calculating the average of the difference between the average accuracy of the two models at $N_{s} { = 1,5,10}$, the average accuracy of the optimized model can be higher than 0.063 in the case of a single domain, and 0.034 in the case of cross-domain. At the same time, it can be seen that because the cross-domain data set is more complex, the average accuracy of the two models is higher in the single-domain case. Moreover, there is a downward trend when $N_{s} = 5$ in the cross-domain case. Considering that the support set and the query set no longer originate from the data set of the same domain in the cross-domain case, it is related to the respective capacity of the support set and the query set.

## Discussion

For this study, we explored the improvement of the training effect of the few-sample regression by optimizing meta-learning, hoping to provide two references for the community. One of them is the difficulty of sample collection and high cost in the field of agricultural irrigation magnetized water and fertilizer. We are committed to solving the limitations of a few samples for prediction, and we have made efforts to optimize the performance of the model. Another point is that the field of few-shot learning in agriculture is not perfect at this stage, and the classification task of sample recognition is mainly, such as the classification of plant leaf diseases. The application of the meta-learning optimization model in the regression task is the follow-up few-shot regression training. Research has a certain paving effect.

We propose a meta-learner LSTM optimized meta-learning optimization model to be applied to the regression prediction of a few samples of PCPMWF. The experimental results compared the LSTM model, MAML model, and Meta-learner LSTM meta-learning optimization model from three aspects: the evaluation criteria of MAE and MAPE, the fitting effect of the true value and the predicted value, and the iterative trend of the loss function. It turns out that no matter from which aspect, the meta-learner LSTM optimization model performs relatively well. Specifically, in the case of few samples, for the MAE evaluation criteria, the meta-learner LSTM optimization model decreased by 0.03% compared with the MAML model, and decreased by 0.2% compared with the LSTM model. For the MAPE evaluation criteria, the meta-learner LSTM optimization model decreased by 0.37% compared to the MAML model, and decreased by 4.16% compared to the LSTM model. In terms of the fitting effect of the true value and the predicted value, it is obvious that the prediction value fit of the meta-learner LSTM optimization model is better than the other two models. The loss value of the meta-learner LSTM optimization model changes significantly after 5 iterations, and there is no obvious trend change when the number of iterations exceeds 10. Compared with the MAML model and the LSTM model, the loss value declines the fastest and steadily, and has good robustness.

Through cross-domain experiments, it is found that although the average accuracy of the model is relatively lower than that obtained from the single-domain data set, the average accuracy of the optimized model proposed in this paper can still reach 0.833, and it is always higher than the MAML model. At the same time, it was found in experiments that the average accuracy of the model in the cross-domain case did not increase with the increase in the number of sample points in the support set. The reason may be related to the capacity allocation of the support set and the query set. In the future experimental work, we can further explore its influence on the model.

At the same time, during the training process, we found that although the proposed model performed well in the prediction effect, the calculation speed decreased significantly. Therefore, in the follow-up, we hope to further conduct multi-factor experimental analysis in terms of parameter adjustment and model structure deployment to improve the promotion performance and prediction efficiency of the few-shot regression prediction model.

The disadvantage of this article is that it uses a self-built datasets and does not fully analyze the subjectivity of experimental measurement. In the future, we will consider using data enhancement and other methods to conduct full verification and comparative analysis more objectively, so as to explore the fact that there are fewer samples. Should we expand the number of samples to fit the model, or should we continue to improve the model to better train a small number of samples. At the same time, this article only selects two models for comparison in the machine learning algorithm, and will further consider adding analysis and verification of related models in the future.

## Conclusion

The application of predicting the properties of magnetized water and fertilizer based on a few samples is of great significance to the field of regression of learning with a few samples. This article focuses on forecasting and analyzing the trend of magnetized irrigation water and fertilizer combined with the method of few-shot learning to provide a reference value for the agricultural field and machine learning research. In this work, we proposed the meta-learning optimization model of meta-Learner LSTM. Meta-Learner LSTM and the gradient descent method complement each other. After multiple updates, it can quickly improve the learning efficiency of the model, which is more accurate than traditional MAML. Its superiority is manifested in theory and experiment. We verify the correctness and robustness of the proposed model through comparative experiments. In the case of using the same dataset, the average absolute percentage error of the meta-learning optimization model of the proposed meta-Learner LSTM is reduced by 0.37% compared with the MAML model, and by 4.16% compared with the LSTM model. Moreover, the loss value of the meta-learning optimization model in the iterative process drops the fastest and steadily compared to the MAML model and the LSTM model. In cross-domain experiments, the average accuracy of the meta-learning optimized model can still reach 0.833, and it is always higher than the MAML model under any circumstances. Cross-domain exploration will also become a major direction of future research.

## Data Availability

The datasets used in this study is available from the corresponding author on reasonable request.
